# Mitochondrial dysfunction in Parkinson’s disease

**DOI:** 10.1186/s40035-016-0060-6

**Published:** 2016-07-22

**Authors:** Qingsong Hu, Guanghui Wang

**Affiliations:** Laboratory of Molecular Neuropathology, Jiangsu Key Laboratory of Translational Research and Therapy for Neuro-Psycho-Diseases and College of Pharmaceutical Sciences, Soochow University, Suzhou, Jiangsu 215021 China; The Second Affiliated Hospital of Soochow University, Soochow University, Suzhou, Jiangsu 215021 China

**Keywords:** Parkinson’s disease, Neurodegeneration, Mitochondrial deficiency, MPTP, Mitochondrial complex I inhibitor, Mitophagy

## Abstract

Parkinson’s disease (PD) is the second most common neurodegenerative disease, which is characterized by loss of dopaminergic (DA) neurons in the substantia nigra pars compacta and the formation of Lewy bodies and Lewy neurites in surviving DA neurons in most cases. Although the cause of PD is still unclear, the remarkable advances have been made in understanding the possible causative mechanisms of PD pathogenesis. Numerous studies showed that dysfunction of mitochondria may play key roles in DA neuronal loss. Both genetic and environmental factors that are associated with PD contribute to mitochondrial dysfunction and PD pathogenesis. The induction of PD by neurotoxins that inhibit mitochondrial complex I provides direct evidence linking mitochondrial dysfunction to PD. Decrease of mitochondrial complex I activity is present in PD brain and in neurotoxin- or genetic factor-induced PD cellular and animal models. Moreover, PINK1 and parkin, two autosomal recessive PD gene products, have important roles in mitophagy, a cellular process to clear damaged mitochondria. PINK1 activates parkin to ubiquitinate outer mitochondrial membrane proteins to induce a selective degradation of damaged mitochondria by autophagy. In this review, we summarize the factors associated with PD and recent advances in understanding mitochondrial dysfunction in PD.

## Background

Parkinson’s disease (PD) is the second common neurodegenerative disease that affects about 1 % of adults over 60 [1]. The motor symptoms of PD are rigidity, bradykinesia, postural instability and resting tremor, which are caused by a progressive loss of dopaminergic (DA) neurons in the substantianigra pars compacta (SNpc) [2]. Besides loss of DA neurons in SNpc, in most familial and sporadic PD, the surviving DA neurons present cytoplasmic and neuritic inclusions named Lewy bodies (LBs) and Lewy neurites that are mainly composed of alpha-synuclein (α-syn), with other proteins in surviving DA neurons [3, 4]. Although the causative factors for DA neuronal loss are still unclear, multiple events contribute to PD pathogenesis, including protein aggregation [5], impairment of the ubiquitin-proteasome pathway [6], oxidative stress [7], mitochondrial dysfunction [8, 9] and neuroinflammation [10–12]. Clinically, most PD cases are sporadic; however, autosomal dominant and recessive familial forms that are resulted from mutations in PD-associated genes have been identified in the past 2 decades. Both environmental and genetic factors can induce mitochondrial dysfunction. Many of the PD-associated gene products are mitochondria-resident proteins or can be translocated to mitochondria upon stimulations. They function in either protecting or damaging mitochondria. Mutations in these genes may result in either loss or gain of function, thereby inducing mitochondrial dysfunction. Importantly, some PD-associated gene products such as PINK1 and parkin are identified as key factors involved in the induction of mitophagy, a cellular process to clear damaged mitochondria. In this review, we will discuss the role of PD-associated factors in mitochondrial dysfunction.

### Evidence of mitochondrial dysfunction in sporadic PD brain and neurotoxin-induced animal model

Mitochondrial dysfunction is tightly associated with PD pathogenesis. The direct evidence of mitochondrial dysfunction in PD came from PD patient brain samples [13, 14]. In SN of PD patients, the mitochondrial complex I activity is significantly decreased [13, 14]. Moreover, a high level of mitochondrial DNA deletion was observed in SN neurons from PD patients [15], suggesting a role of mitochondrial dysfunction in PD. Furthermore, decreases of peroxisome proliferator-activated receptor gamma coactivator 1-alpha (PGC-1α, a co-activator important for mitochondrial gene expressions) and PGC-1α-regulated mitochondrial genes were observed in DA neurons in PD [16, 17]. These data suggest the presence of defects in mitochondrial function and biogenesis in PD brain.

The direct linkage of mitochondrial dysfunction with PD came from the discovery of 1-methyl-4-phenyl-1,2,5,6-tetrahydropyridine (MPTP), a neurotoxin that induces PD symptoms in drug-abused patients in 1983 [18]. Soon, the neurotoxicity of MPTP was confirmed in primate and rodent models [19–21]. Later, the inhibitory effects of MPTP on mitochondria were identified [22–24]. Now, it is well known that the neurotoxicity of MPTP arises from its toxic metabolite 1-methyl-4-phenylpyridinium (MPP^+^). MPTP is converted by monoamine oxidase in astrocytes to form 1-methyl-4-phenyl-2,3-dihydropyridinium, which is rapidly converted to MPP^+^ [25]. MPP^+^ is released from astrocytes through the organic cation transporter 3 and taken up by DA neurons through the dopamine transporter [26]. MPP^+^ accumulates in mitochondria to interfere the electron transport chain by inhibiting complex I, leading to ATP depletion and reactive oxygen species (ROS) production [24, 25]. Induction of PD by the inhibition of complex I is also evidenced from studies using rotenone and paraquat, two pesticides with similar structure as MPTP, that inhibit complex I [27]. ROS production by inhibition of complex I is a key mechanism for DA neuronal damage as DA neurons are susceptible to oxidative stress due to autoxidation of DA during catabolism [28]. The generation of ROS induces the damage of complex I and III, and oxidation of proteins on mitochondria and in cytoplasm, leading to mitochondrial dysfunction [29, 30]. The increased oxidative stress overloads the ubiquitin-proteasomal system (UPS), resulting in the accumulation of damaged and misfolded proteins [30, 31]. Importantly, administration of complex I inhibitors induces loss of DA neurons and enables animals to develop the clinical features of PD, which has been commonly used for producing laboratory PD model mimicked sporadic PD for addressing the mechanism and exploring the therapeutics [32, 33].

### Autosomal dominant PD gene products in association with mitochondrial dysfunction in PD

#### *SNCA* (*PARK1*)

*SNCA* (*PARK1*) that encodes α-syn was the first gene identified to be associated with familial PD [34, 35]. α-syn is a major component of cytoplasmic inclusions (LBs) in survived DA neurons in PD brain [36, 37]. α-syn is highly enriched in presynaptic terminals [38]. It interacts with synaptic vesicles and regulates vesicle trafficking and endocytosis [39]. Although the neuronal toxicity of α-syn induces a wide range of cellular dysfunctions in cytoplasm, such as oxidative stress, synaptic transport, UPS impairment and autophagy dysfunction [40–42], the linkage between α-syn and mitochondrial dysfunction has been recently identified. α-syn has a mitochondrial localization, although the majority of α-syn is soluble in cytoplasm [43–45]. Most recently, α-syn is identified to be located at the mitochondria-associated membranes that connect mitochondria and endoplasmic reticulum [45]. Overexpression of pathogenic α-syn (A53T or A30P) induces mitochondrial fragmentation, probably through inducing cleavage of dynamin-like 120 kDa protein (OPA1), a negative regulator of mitochondrial fragmentation [45]. In transgenic mice, the pathogenic α-syn (A53T) inhibit complex I activity and induce mitochondrial degeneration [46]. The transgenic mice present axonal degeneration, neuronal cell death and cytoplasmic inclusions positive for α-syn and nitrated α-syn, presenting pathological features as PD brains [46]. Thus, above studies provide evidence that α-syn has effects on mitochondria, besides its indirectly influencing mitochondrial function by the induction of oxidative stress.

#### *LRRK2* (*PARK8*)

Mutations in *LRRK2* (*PARK8*) are associated with autosomal dominant PD [47–49]. The frequency of mutation G2019S was reported in 5-6 % of autosomal dominant PD patients [50, 51] and even near 1 % of sporadic PD patients without a known family history of the disease [52]. LRRK2 (leucine-rich repeat serine/threonine-protein kinase 2) is located in mitochondria, cytoplasm and nucleus [53]. The kinase activity of LRRK2 G2019S is increased [53]. The mitochondrial membrane potential and ATP level are decreased but mitochondrial elongation is increased in fibroblasts from PD patients harboring *LRRK2 G2019S* mutation [54]. LRRK2 G2019S increases uncoupling protein level to depolarize mitochondrial membrane potential [55]. LRRK2 interacts with dynamin-related protein 1 (DRP1), a mitochondrial fission protein [56]. Inhibition of LRRK2 activity increases mitochondrial ROS production, DRP1 mitochondrial translocation and mitochondrial fission, suggesting an involvement of LRRK2 in the regulation of mitochondrial dynamics and oxidative stress [57].

#### *CHCHD2* (*PARK22*)

Recently, the association of mitochondrial dysfunction with PD is further evidenced by the identification of a missense mutation in *CHCHD2*, an autosomal dominant gene associated with late-onset PD in a Japanese family [58] and a risk factor for sporadic PD [59]. *CHCHD2* (*PARK22*) encodes coiled-coil-helix-coiled-coil-helix domain-containing protein 2 (CHCHD2), a protein originally identified as a transcription factor that binds to oxygen responsive element of *COX4I2*, a gene encoding cytochrome c oxidase (COX) subunit 4, isoform 2 that regulates cytochrome c oxidase activity [60]. As a transcription factor, CHCHD2 transactivates the nuclear encoded COX4I2 in nucleus [61]. However, it is also a mitochondrial intermembrane space-resident protein bound to COX and regulating COX activity [61]. Decrease of CHCHD2 level results in decreases of COX activity and mitochondrial membrane potential, and increases of ROS production and mitochondrial fragmentation [61]. Moreover, CHCHD2 functions in mitochondria to anti-apoptosis through its interacting with Bcl-xl to inhibit the oligomerization and mitochondrial accumulation of Bax [62].

### Autosomal recessive PD gene products in association with mitochondrial dysfunction in PD

#### *PARKIN* (*PARK2*)

Three of autosomal recessive PD genes *PARKIN* (*PARK2*), *PINK1* (*PARK6*) and *DJ-1* (*PARK7*) are tightly associated with mitochondrial dysfunction in PD. *PARKIN* is the first recessive gene identified to be associated with autosomal recessive juvenile Parkinsonism in a Japanese family [63], just 1 year after the discovery of *SNCA* (α-syn). Mutations in *PARKIN* have been found in patients of different ethnicity and account for about half of known cases of autosomal recessive PD [64, 65]. Parkin, the *PARKIN* gene product, is a RING finger containing E3 ligase [66]. A logical hypothesis is that loss of parkin function will result in the accumulation of its substrates that may be toxic for DA neurons [67]. However, up to date, most identified substrates of parkin are not exclusively expressed in DA neurons or accumulated in PD [68]. And the pathological LBs are absent in PD cases with *PARKIN* mutations. It is possible that parkin-mediated non-degradation signal also plays roles in PD as it can ubiquitinate substrate through either K63- or K48-linked ubiquitin chains [69]. One of the parkin substrates is PARIS (Zinc finger protein 746), a major repressor of PGC-1α [70]. Parkin ubiquitinates PARIS and regulates its expression level. In *PARKIN* knockout mice and PD brains, the PARIS levels are increased but PGC-1α levels are decreased [70]. As PGC-1α is a central regulator for nuclear and mitochondrial encoded gene expressions, the mitochondrial protein expressions are decreased with the decrease of PGC-1α in PD brains [71]. Thus, parkin may regulates mitochondrial biogenesis by its indirectly infleuencing PGC-1α level. Thus, the accumulation of PARIS in PD brain reflects loss of parkin E3 ligase activity-induced impairment of protein degradation and provides an explanation of mitochondrial dysfunction in PD.

#### *PINK1* (*PARK6*)

PINK1 is a mitochondrial serine/threonine protein kinase encoded by *PINK1* gene which mutations cause an autosomal recessive form of PD [72]. PINK1 is known as a parkin upstream factor that accumulates on mitochondria upon depolarization of mitochondria and recruits parkin onto mitochondria [73]. As a mitochondrial protein, PINK1 has multiple roles in mitochondria, including mitophagy [73], mitochondrial traffic [74], mitochondrial dynamics [75] and complex I activity [76]. Depolarization of mitochondria induces PINK1/parkin to associate with Miro, a mitochondrial out membrane protein that recruits kinesin to the mitochondrial surface [74]. PINK1 phosphorylates Miro to induce a parkin- and proteasomal-dependent degradation of Miro, thereby releasing kinesin from mitochondria, leading to an inhibition of mitochondrial motility [74], which may be an initial quarantining step prior to mitophagy [74]. PINK1/parkin pathway also affects mitochondrial dynamics. Both mitochondrial fusion- and fission-proteins, such as mitofusin (Mfn) [77, 78] and DRP1 [79], are parkin substrates that are ubiquitinated by parkin. Phosphorylation of Mfn2 by PINK1 is required for Mfn2 interaction with and ubiquitination by parkin [78]. PINK1 deficiency causes defect of complex I, mitochondrial depolarization and increased sensitivity to apoptotic stress [80]. The deficiency of complex I by loss of PINK1 can be rescued by wild type PINK1, but not PD-related mutant PINK1 [80]. Interestingly, the impaired mitochondrial respiration is presented in the striatum but not in the cerebral cortex in young *PINK1* knockout mice, suggesting a specific involvement of PINK1 in DA circuitry [81]. Recently, NADH dehydrogenase [ubiquinone] 1 alpha subcomplex subunit 10 (NdufA10), a complex I subunit, was identified to be phosphorylated at S250 dependent on PINK1 [76]. Loss of phosphorylation of S250 in NdufA10 was observed in *PINK1* knockout mice [76]. Introduction of S250D NdufA10 into *PINK1* deficient cells or mutant fly restores complex I activity and membrane potential [76], suggesting a critical role of PINK1 in regulating complex I activity through NdufA10 phosphrylation.

#### *DJ-1* (*PARK7*)

*DJ-1* is another PD gene which missense or deletion mutations are associated with autosomal recessive PD [82]. DJ-1 is a multifunctional protein involved in many cellular functions [83], including transcriptional regulation [84, 85], anti-oxidative stress [86–88], chaperone activity [89] and protecting mitochondria [87]. DJ-1 protects cells against ROS by self-oxidation at C106 [86]. Although DJ-1 lacks mitochondrial targeting sequence and is mainly cytosolic, it can be translocated onto mitochondria against oxidative stress-induced cell death [87]. However, pathogenic forms of DJ-1, such as L166P and M26I, are localized on mitochondria and sensitize cells to oxidative stress [82, 90]. DJ-1 binds to complex I subunits and loss of DJ-1 decreases complex I activity [91], suggesting that DJ-1 has impact on complex I. Interestingly, similar mitochondrial phenotype can be observed in *PINK1*- or *DJ-1*-deficient cells and mitochondrial defects in *DJ-1*-deficient cells can be rescued by parkin or PINK1, although PINK1/parkin pathway seems functioning in parallel to, rather than downstream of, DJ-1 pathway [92].

#### *HTRA2/OMI* (*PARK13*)

The *HTRA2/OMI* (*PARK13*) gene product HtrA2/OMI (HtrA serine peptidase 2, refer to OMI later) is a mitochondrial serine protease that was first identified as a mammalian homologue to bacterial heat shock endoprotease HtrA and named as OMI [93]. It is released from mitochondria to cytosol to cleave XIAP in response to apoptotic stimuli, which induces apoptosis [94]. In 2005, it was found that loss of OMI protease activity is associated with PD [95]. *mnd2* (motor neuron degeneration 2) mice which harbor protease-deficient OMI S276C mutants, and *OMI*-knockout mice present motor abnormalities similar to PD, with the progressive neurodegeneration in some brain regions, especially in striatum [96]. Loss of OMI protease activity leads to mitochondrial dysfunction. The cells from *mnd2* or *OMI* knockout mice increased susceptibility of mitochondrial membrane permeabilization, decreased mitochondrial membrane potential, and reduced mitochondrial density [96–98]. In *OMI* knockout mouse embryonic fibroblasts, the damage and mutation of mitochondrial DNA are increase [99]. Interestingly, PINK1 interacts with OMI and facilitates OMI phosphorylation, which contributes to increased resistance of cells to mitochondrial stress [100]. Moreover, in brains from PD patients with *PINK1* mutaions, the phosphorylation of OMI is decreased, further suggesting that PINK1 acts on the upstream of OMI in a mitochondrial stress sensing pathway in PD.

#### *PLA2G6* (*PARK14*)

The *PLA2G6* (*PARK14*) gene encodes an 85-kDa calcium-independent phospholipase A_2_β (PLA2G6) that hydrolyses the sn-2 acyl chain of glycerophospholipids to release free fatty acids from phospholipids [101]. *PLA2G6* gene mutations cause PLA2G6-associated neurodegeneration (PLAN), including infantile neuroaxonal dystrophy [102] and adult-onset dystonia-parkinsonism [103, 104]. PLA2G6 is distributed in cytosol and membrane associated compartments, but mostly in mitochondria [105]. In cells, overexpression of PLA2G6 protects cells from staurosporine-induced apoptosis through stabilizing mitochondrial membrane potential, reducing mitochondrial reactive oxygen species production [105]. In *Drosophila*, loss of *iPLA2-VIA*, the *Drosophila* orthologue of *PLA2G6*, leads to age-dependent locomotor deficits and neurodegeneration [106]. The flies lacking *iPLA2-VIA* display severe mitochondrial degeneration with decreases of mitochondrial membrane potential and ATP production [106]. In *PLA2G6* knockout mice, abnormal mitochondria with multiple morphological changes are presented in the anterior horns spinal cord [107]. Most interestingly, in *PLA2G6* knockout mice as well as *PLA2G6* knockdown cells, α-syn levels are increased [108]. The immunoreactivity of S129-site phosphorylated α-syn is strongly presented in neuronal granules which are labeled with mitochondrial outer membrane 20 kDa protein (TOM20) in *PLA2G6* knockout mice [108], suggesting an accumulation of α-syn on damaged mitochondria. In PLAN brains, α-syn labeled small inclusions are colocalized with TOM20, which may develop to LBs [108], further suggesting a role of PLA2G6 in mitochondrial dysfunction and LB formation.

### Mitophagy

Mitochondrial dysfunction is a key pathological change in PD. The only way to clear the damaged mitochondria is mitophagy, a cellular process for a selective degradation of mitochondria by autophagy [109]. The role of PINK1/parkin in mitophagy has been extensively studied after the discovery of PINK1/parkin selectively driving damaged mitochondrial degradation [110]. The early hints of PINK1/parkin on mitochondrial homeostasis came from studies using *Drosophila* model [111–113]. *Drosophila park* null flies present prominent mitochondrial damage in muscle [111]. Similar phenotype was observed in *pink1* null flies [112, 113]. Overexprsssion of parkin in *pink1* null flies rescues mitochondrial phenotype, but overexpression of PINK1 does not rescue the phenotype in *parkin* null flies, suggesting that parkin functions in the downstream of PINK1 [112, 113].

The role of PINK1/parkin in mitophagy was identified the study that parkin is selectively recruited to damaged mitochondria to drive mitochondrial degradation after the treatment of carbonyl cyanide m-chlorophenylhydrazone (CCCP), a mitochondrial uncoupler that induces mitochondrial depolarization [110]. The recognition of mitochondria for autophagic degradation needs either mitophagy receptor or ubiquitinated protein on mitochondrial membrane. The substrates of autophagy need to be interacted with phosphatidylethanolamine-conjugated LC3 (microtubule-associated protein light chain) that is anchored on phagophore [114, 115]. Three mammalian mitophagy receptors are recently identified, including Nix (BCL2/adenovirus E1B 19 kDa protein-interacting protein 3-like) [116], FUNDC1 (FUN14 domain-containing protein 1) [117], and BCL2L13 [118], that are located on the outer mitochondrial membrane (OMM) and able to interact directly with LC3 to induce mitophagy. The induction of mitophagy dependent on PINK1/parkin pathway is mediated by ubiquitination of mitochondria, on which the ubiquitin chains are recognized by autophagic adaptors that interact with LC3 through LC-interaction region (LIR) to link LC3-conjugated phagophore and ubiquitinated mitochondria [119].

Parkin ubiquitinates OMM proteins with K48- and K63-linked ubiquitin chains that play roles in parkin-dependent mitophagy [120]. The parkin-dependently ubiquitinated mitochondria are recognized by autophagic receptor p62 [121] or NBR1 [120, 122] for mitophagy. Interestingly, parkin induces a degradation of OMM proteins such as Mfn1, Mfn2, TOM70 and others, which is independent on autophagy but dependent on UPS [120, 123]. Degradation of OMM proteins by UPS promotes mitophagy, probably by influencing mitochondrial motility [120, 123]. Deubiquitination by deubiquitinases (DUBs) are also involved in the regulation of mitophagy. The ubiquitin-specific protease (USP) 15 decreases parkin-attached mitochondrial ubiquitin chains to interfere parkin-driven mitophagy [124]. The mitochondrial DUB, USP30, also removes ubiquitin chains on mitochondria to block parkin-induced mitophagy [125]. Knockdown of *USP15* or *USP30* improves the phenotype of parkin- or PINK1-deficient flies, suggesting a functional interaction between mitochondrial ubiquitination by parkin and deubiquitination by DUBs.

Recently, the kinase activity of PINK1 and its role in the clearance of damaged mitochondrial are well documented and reviewed [126, 127]. PINK1 phosphorylates parkin at S65 to activate parkin and to induce parkin recruitment onto mitochondria [128]. PINK1 also pohosphorylates ubiquitin at S65, which activates parkin E3 ligase activity [129–131]. It seems that the phosphorylation of ubiquitin chains on damaged mitochondria by PINK1 is prior to and promotes parkin recruitment onto mitochondria [129, 130]. Mitochondrial damage induces accumulation of PINK1 that phosphorylates and activates parkin and ubiquitin. Meanwhile, mitochondrial damage activates serine/threonine-protein kinase TBK1, a kinas that phosphorylates autophagic adaptor optineurin, Nuclear domain 10 protein NDP52 and p62, and induces them recruitment to damaged mitochondria, leading to activation of mitophagy [132].

## Conclusions

Evidence from PD patients and animal models indicate a linkage between mitochondrial dysfunction and PD pathogenesis. Environmental and genetic factors contribute to mitochondrial dysfunction in PD. One of common defects in PD patients and PD model is the deficiency of complex I. Recent findings indicate that PINK1 and parkin are involved in mitophagy. PINK1 can be accumulated on damaged mitochondria to recruit parkin onto mitochondria, resulting in ubiquitination of OMM proteins and induction of mitophagy. Loss of PINK1 or parkin leads to a failure in the clearance of damaged mitochondria, thereby inducing DA neurons susceptible to stresses. However, it is still unclear why the damaged mitochondria are not successfully cleared in sporadic PD patients or in neurotoxin- or genetic factor-induced animals that harbor wild type PINK1 and parkin. It is also unclear why loss of mitochondrial membrane potential does not induce PINK1 accumulation on mitochondria to promote mitophagy for the clearance of damaged mitochondria *in vivo*, although it induces mitophagy in cellular models. It is of help to identify the factors that influence PINK1 activity and accumulation on mitochondria and that affect or block PINK1 downstream factor activation in both cellular and animal models.

## Abbreviations

α-syn, alpha-synuclein; CCCP, carbonyl cyanide m-chlorophenylhydrazone; CHCHD2, coiled-coil-helix-coiled-coil-helix domain-containing protein 2; COX, cytochrome c oxidase; DA, dopaminergic; DRP1, dynamin-related protein 1; DUB, deubiquitinases; FUNDC1, FUN14 domain-containing protein 1; HtrA2/OMI, HtrA serine peptidase 2; LBs, Lewy bodies; LC3, microtubule-associated protein light chain; LIR, LC-interaction region; LRRK2, leucine-rich repeat serine/threonine-protein kinase 2; Mfn, mitofusin; *mnd2*, motor neuron degeneration 2; MPP^+^, 1-methyl-4-phenylpyridinium; MPTP, 1-methyl-4-phenyl-1,2,5,6-tetrahydropyridine; NDP52, Nuclear domain 10 protein; NdufA10, NADH dehydrogenase [ubiquinone] 1 alpha subcomplex subunit 10; Nix, BCL2/adenovirus E1B 19 kDa protein-interacting protein 3-like; OMM, outer mitochondrial membrane; OPA, dynamin-like 120 kDa protein; PARIS, zinc finger protein 746; PD, Parkinson’s disease; PGC-1α, peroxisome proliferator-activated receptor gamma coactivator 1-alpha; PLA2G6, 85-kDa calcium-independent phospholipase A_2_β; PLAN, PLA2G6-associated neurodegeneration; ROS, reactive oxygen species; TOM20, mitochondrial outer membrane 20 kDa protein; UPS, ubiquitin-proteasomal system; USP ubiquitin-specific protease
